# Cases of Intermittent Fever

**Published:** 1856-09

**Authors:** W. A. Peck

**Affiliations:** Berwick, Pennsylvania


*Cases of Intermittent Fever.* By W. A. Peck, M. D., Berwick, Pennsylvania.

Case I.† Mary W——, a hale German girl, of 18 years, on the 25th of August, 1855, had an attack of uncomplicated remittent fever, which readily yielded to the usual remedies. This fever was succeeded, after a week's convalescence, by paroxysms of a retarding quotidian intermittent. These she continued to

*It may be mentioned in passing, that cane sugar was also converted into grape sugar in St. Martin's stomach. In the product of digestion of calves' feet jelly, glucose was distinctly recognisable. The gelatine was unchanged.

† Reported in the Boston Med. and Surg. Journal, in Vol. LIII. No. 16.

have, presenting nothing at all remarkable until the fifth paroxysm, when I was called in to witness its singularity. I found her with her right side, arm and leg, shaking most valorously, *while her left side and limbs presented their usual phenomena.* The right side was extremely cold, with *cutis anserina;* small, quick and accelerated pulse, with the other usual characteristics of the ague. The median line very accurately defined the extent of the morbid action. There was, however, a shading off of the coldness of the right, to the natural warmth of the left side.

The skin of the left side presented its usual temperature and moisture; the pulse was much fuller than on the right side. The motor and sensory functions, though perverted on the right, were on the left side perfectly normal. There was slight tenderness in the right hypochondrium, and in the epigastrium, which was the only accompanying symptom discoverable worthy of note. The chill lasted about two hours, when it was regularly succeeded by the hot stage. The right side went through the whole succession of phenomena, as regularly as though both sides were companions in misfortune.

The pulse of the left side partook, of course, of the acceleration of the right, but had not its fulness and tension. The temperature of the skin was slightly elevated, which provoked a moderate perspiration. The fever of the right side went off in the course of four hours, with a profuse sweat. She never has presented any lateral derangement of the cerebro-spinal axis before or since, though she has had the ague repeatedly, and has uniformly brought "all fours" into service.

Case II. Mrs. O——, a plethoric, active woman, of 30 years, had been subject to intermittent fever, either in its primary form, or, as consecutive to remittent fever, from her childhood. About five years ago, she had a violent attack of remittent fever, which was not arrested until the third paroxysm, which resulted in a *partial anæsthesia of the right side.* I am unable to get a satisfactory history of this paroxysm from her attending physician, further than that the "nervous system seemed to be entirely overwhelmed." Since this time, she has repeatedly suffered from attacks of intermittent and remittent fever, and with an uniformity of results. Her paroxysms now were very like the one described in case first, *i. e.*, the side normally innervated were not only colder in the cold stage, but hotter in the pyrexial, and perspired more freely in the sweating stage. She has since died, in the third paroxysm of a bilious fever, which, from the beginning, presented no evidence of disease of the right side whatever, save the anæsthesia. The two first paroxysms were similar to the latter, save in the completeness of the paralysis. Aside from the peculiarity already mentioned, there were no symptoms of an anomalous character. It is to be regretted that a *post-mortem* was not permitted.

Case III. Jas. McN——, an Irish laborer, 45 years of age; suffered repeated attacks of *fever and ague.* In the midst of a succession of these, he was seized with a spinal meningitis of the lumbar region, which afterwards assumed a chronic form, when, by degrees, it resulted in complete paralysis of the inferior extremities.

Before, however, the paralysis became complete, he had an attack of *ague*, which, like case second, affected the parts normally innervated more than the paralytic members. After the complete palsy of the inferior members, neither the cold nor the hot stage had any impression whatever on them. After a tonico-alterative course of treatment, with cold effusions, and a succession of blisters, issues and setons, the man recovered, both from his spinal affection and paralysis; and ever after enjoyed all the benefits to be derived from having the ague on "all fours."

Case IV. Another case occurs to my mind of precisely similar character. The subject was a feeble girl, of 11 years of age, who came under charge in August, 1855, for treatment of spinal meningitis in the lumbar region, and an approaching paralysis. Like case 3d, she had ague before the accession of palsy, and of the usual type; like him, too, had ague when the paralysis was incomplete and complete, and finally, after she had recovered from it, and with the same results.

Case V. This was one of a Swedish collier, aged 40 years; robust and hearty, until by a fall he injured his back, which was subsequently followed by effusion and consequent paralysis of the inferior extremities. The sequel of this case is so exactly similar to the results of the preceding cases, both in the termination of the case and its behavior in the presence of an intermittent, that it is unnecessary for me to add more.

The foregoing cases, though briefly described, are sufficiently explicit, I trust, to accomplish the object I have in view in their record. These cases have all occurred under my own observation, and I doubt not that many practitioners, who have had much to do with miasmatic fevers, could add many more.

The pathological phenomena presented in the above cases, afford us the most positive evidence that the proximate cause of paroxysmal pyrexiæ, is the cerebro-spinal axis laboring under the influence of miasma. Hence it is entirely unnecessary for us to formally notice the wild guesses of Willis, Deleboe, Borrelli, and Torti, as to periodical fermentations, acrimonious secretions of the pancreas, &c.; or for a moment entertain the views of Boerhaave and Stoll, or of Selle and Frank as to the unknowable action of the nervous system in the production of agues. It is entirely unnecessary for us to review the characteristics of fever for a satisfactory explanation of the *modus operandi* in producing it. Nor, indeed, need the doctrines of Broussais, Boisseau, Mongellaz, Elliotson, and others, detain us for investigation; for if the vivisection of the *portio dura* demonstrate that nerve to govern the general movements of the face, as the excito-motor acts of a decapitated animal prove the spinal cord to be an independent centre, or the division of that centre shows that its integrity of structure is positively essential to the normal action of the sensory-motor apparatus, then certainly we can as confidently assert, from the testimony of the above cases, that paroxysmal fevers of a malarious origin are primarily affected, modified and controlled by the spinal marrow. For, if an arrest of the normal action of a part of the cord also arrests phenomena which persist in parts normally innervated, certainly the conclusion is unavoidable that the *cord* is the instrument through which malaria acts.

Indeed, an examination of the disposition and physiognomy of this class of fevers is sufficient to convince us that these cases are not anomalous freaks of diseased action, but precisely what we might, *a priori*, expect. The first great character which distinguishes them from the other of the *febres*, *i. e.*, *periodicity*, suggests a community of origin with the same phenomenon witnessed in *neuralgia*, *epilepsy*, *hysteria*, *insanity* and other affections, of whose *habitat* there is no question, as well as with the divers physiological periodical cycles, which unquestionably acknowledge a neurotic origin. The fact that all cases of periodicity are produced by successional modifications of the nutritive function, affords no valid argument against this conclusion; since these very organic changes are wrought in the organs which manifest these phenomena, and constitute the proximate cause. And it matters not whether these changes were produced by the operation of the "general vital stimuli," or by the agency of *materies morbi*, as long as the instrument continues to be the *cerebro-spinal axis*.

The premonitory symptoms of fever, *i. e.*, pain in the head and back, feelings of weariness, stretching, yawning, &c., very generally point to the spinal axis as the seat of disturbance. In the *rigors* of the "cold stage," we have another example of sensory motor derangement; for the sensation of cold is by no means uniformly associated with actual coldness of the surface, but on the contrary, it is often quite the reverse. In the "pernicious" form of fever, one of the most striking of its phenomena is the doleful lamentation concerning the increased heat, whereas, the surface may be intensely cold. The somewhat characteristic chilly, longitudinal lines, certainly are as capricious as any of the protean *didoes* of hysteria. Besides these, we have the neuralgic pains of the head, back, loins and extremities; occasionally drowsiness, stupor and coma, or the mind morbidly excitable, confused, dejected, or by times wandering. There are other symptoms of the cold stage which depend upon the actually depressed state of the organic functions; but as they stand in no uniform relation to those above mentioned, they must be presumed to be consecutive sequences of the proximate cause.

There are likewise symptoms of the hot and sweating stages, which cannot be attributed to *pyrexia*, as they stand in no uniformly commensurate relation with it, only such as both classes would necessarily bear to the intensity of the cause. Such are pain in the head, back and limbs; convulsions in children; delirium, coma, spasms, &c., &c. The phenomena presented by *pernicious* intermittents also plainly set forth morbid innervation as their proximate cause.

Thus, in uncomplicated paroxysmal fevers, we have sufficient evidence for drawing the conclusion that, at all events, the spinal cord is an instrument through which miasma acts to produce a portion of the symptoms peculiar to the disease. But fortunately we can not only with certainty assert that the spinal cord is affected in these fevers, but that it has a most controlling influence over the nature, severity, and duration of the paroxysm; and not only so, but that its normal integrity of structure and function are positively essential, in order to the production of a complete paroxysm of fever.

The queries very naturally arise, whether the cerebro-spinal nervous system refuses to respond to the reaction of the organic system under the influence of miasm; or, is this system primarily affected, and the action of the sympathetic consecutive; or, are both systems primarily involved in the production of the various phenomena of fevers? That the organic system is concerned in the production of fevers, we must freely allow. That both systems are not primarily affected, must also be sufficiently evident, from the fact, that in paralysis of the sensory-motor apparatus, the parts thus normally innervated present none of the phenomena of fever; whereas, were this the case, it would be difficult to conceive the reason for the absence of derangements in such organs and functions as the sympathetic is known to be able to control. Hence the question resolves itself into this, which system of nerves is dependent for its action in the production of intermitting fevers upon the other for the essential condition of its action?

Now it must be apparent, that were the sympathetic nerves primarily affected, that in case of paralysis of the spinal medulla, there would certainly be an absence of the *perverted sensations* of cold and heat, of neuralgic pains, and of the various other derangements of the sensory-motor function; but there could not, by any known possibility, be an absence of actual coldness in the cold stage, depression of the circulation and general embarrassment of the organic functions; nor of actual heat in the hot stage and fever in its access; nor of sweating in its appropriate stage, since these very phenomena are, to a great extent, under the governance of this system. That such is the case, the evidence already cited abundantly proves; and it furthermore compels us to admit the subordination of the organic to the cerebro-spinal system in the production of miasmatic fevers. Or, that the spinal nerves are primarily affected, and that the ganglionic derangements are consecutive to, and dependent on the reactions of the cerebro-spinal axis.

To this view of the *modus operandi* of *miasma*, it has been objected, that the spinal system of nerves does not supply at all (or but partially) nervous influence to organs especially affected; as the heart, bloodvessels, the secretory viscera, lungs, &c. Granted; but does not the ganglionic system do all this? If so, it is all our theory requires! Again, it is said, that the chief avenues to the system open to the invasion of exciting causes, are the organs of sense and the cutaneous surface; and consequently, if this were the system primarily affected, these causes either could not gain admission, or else the phenomena of intermittents would be referred to these organs! A sufficient reply to this objection is simply the expression of the fact, that the properties of agents capable of exciting the organs of sense, and centrically affecting the spinal cord, are neither identical, nor yet similar. Thus, strychnia undoubtedly expends its energies centrically upon the spinal marrow, which would be a great mistake of the nervous system, if the above doctrine of the necessarily excentric action were true.

Neither is this theory materially affected by the assertion that the total annihilation of the cerebral functions would not produce death as soon as miasmatic action is known to do in some cases. Now it is worth while to recollect, not only that we do not claim paralysis to be the proximate cause of fever, but the fact, that to our senses, the impression produced upon one system of nerves is instantaneous with its repetition on the other. Again, it is claimed, that the cerebro-spinal nerves can exert no influence over the circulatory system; the production of animal heat, the secernent functions; nor yet over the processes of assimilation and nutrition; *ergo* this system of nerves cannot be the only system primarily affected. But again, I must insist upon the consecutive action of the sympathetic nerves as an explanation for the phenomena peculiar to these functions in fever.

But the most potent objection that has been urged against this theory is, that the normal influence of the spinal cord over the organic nerves is insufficient to warrant the conclusion that when under the influence of malaria, it can so far control the last named system as to produce the symptoms attributed to it. This objection, if founded in fact, would successfully invalidate our theory; but the evidence now adduced, shows the inference to be unfounded, and hence speaks in more positive and authoritative terms than any man's *opinion*, from the present state of our knowledge, has a right to assume.

Nor is intermitting fever an isolated example of this influence over the sympathetic system. An error of diet, teething, worms, passage of a catheter, and many other irritating agencies, have all and each raised a very noticeable commotion throughout the economy.

